# Changes in Trauma-based Intrusive Memory Characteristics Associated with Repetitive Transcranial Magnetic Stimulation (rTMS) for Depression: A Daily Diary Study

**DOI:** 10.1007/s11126-025-10156-4

**Published:** 2025-05-06

**Authors:** Amalia Badawi, Zachary Steel, Kris Rogers, Nalin Wijesinghe, David Berle

**Affiliations:** 1 Graduate School of Health, University of Technology, Sydney, Australia; 2 School of Psychiatry, University of New South Wales, Sydney, Australia; 3 St John of God Healthcare, Richmond Hospital, Sydney, Australia; 4 School of Population Health, University of New South Wales, Sydney, Australia; 5 The George Institute for Global Health, University of New South Wales, Sydney, Australia; 6 South Coast Private Hospital, Wollongong, Australia; 7 School of Medicine and Psychology, Australian National University, Canberra, ACT 2601 Australia

**Keywords:** Intrusive memories, Trauma, Re-experiencing symptoms, Transcranial Magnetic Stimulation, Depression

## Abstract

The present study investigated whether repetitive transcranial magnetic stimulation (rTMS) for depression reduced trauma-based intrusive memory frequency and associated pathological characteristics such as intensity and distress. Traumatic stress symptoms and general psychopathology outcomes were also investigated. Inpatients at a psychiatric hospital (*N* = 25) referred for 20-sessions of rTMS for depression completed daily ratings of trauma-related intrusive memory frequency and characteristics. Linear mixed models and repeated measures t-tests were used to analyze the course of change for intrusive memories. Effect sizes and reliable change were also analyzed. Non-linear decreases for intrusive memory frequency and associated characteristics were reported over the course of treatment, with the changes from Days 0 to 7 being sustained at Day 20. Large effect sizes were reported for measures of traumatic stress, and re-experiencing, depression, and stress symptoms. Reliable change was most commonly indicated for measures of traumatic stress and general mental health. Limitations of the study include the lack of a control group and small sample size. To the best of our knowledge, this study is the first to use a within-subject daily-sampling design to monitor intrusive memories, in an inpatient setting, within the context of rTMS treatment. Routine rTMS for depression appears beneficial for reducing trauma-based intrusive memories as well as PTSD symptoms more generally. The present study demonstrates clinical viability of extending routine rTMS protocols used for depression to trauma-based intrusive memories and associated symptomatology.

**Supplementary Information:**

The online version contains supplementary material available at 10.1007/s11126-025-10156-4.

## Introduction

Intrusive memories are a characteristic feature of psychopathology following trauma exposure and have been shown to predict severity and onset of posttraumatic stress disorder (PTSD); [1, 2]. Globally, trauma exposure rates are estimated to be 70% [3], with lifetime prevalence rates of 7–9% indicated for PTSD [4, 5]. Although trauma-focused psychotherapy targets intrusive symptoms, only moderate gains from these treatments are reported for approximately one-third of recipients, with another third continuing to meet diagnostic criteria post-treatment [6]. These outcomes highlight the need for ongoing investigations focused on reducing pathology following trauma exposure.

Several studies have examined the use of transcranial magnetic stimulation (TMS) as a treatment for anxiety and trauma-related disorders, with promising results across different meta-analyses. Cirillo et al. [7] conducted a systematic review and meta-analysis to assess the efficacy of TMS in conditions like generalized anxiety disorder (GAD) and post-traumatic stress disorder (PTSD). Their findings indicated that TMS, particularly when applied to brain regions such as the prefrontal cortex, produced moderate-to-large effects in reducing symptoms of these disorders. Similarly, the meta-analysis conducted by Harris and Reece [8] found that TMS significantly alleviated PTSD symptoms, with no statistically significant differences observed between stimulation of the left versus right dorsolateral prefrontal cortex (DLPFC). Moreover, high-frequency stimulation protocols were associated with the most pronounced therapeutic effects.

The neuromodulation of prefrontal brain structures via rTMS is thought to hold promise for treating PTSD, given the disorder’s impact on both superficial and deep brain structures, as evidenced by neuroimaging studies [9]. Stimulation of the dorsolateral prefrontal cortex (DLPFC) may exert therapeutic effects in PTSD by modulating fronto-limbic circuits. Through its connections with the ventromedial prefrontal cortex, DLPFC activation can enhance top-down regulation of the amygdala, potentially reducing the hyperactive threat responses characteristic of PTSD [10–12]. A review by Karsen et al. [13] noted that rTMS is frequently applied to the DLPFC due to its role in the affect regulation network, which encompasses critical structures related to PTSD, including the amygdala and hippocampus. This is significant because this specific protocol aligns with treatment methods for Major Depressive Disorder (MDD), which has the most substantial evidence base compared to other mental health conditions [14, 15].

Of the studies that have assessed trauma symptoms following high frequency rTMS applied to the LDLPFC [16–18], only Boggio et al. [16] reported on the re-experiencing cluster in PTSD, which includes intrusive memories [4]. Boggio et al. compared three rTMS conditions that involved 20 Hz applied for 10 sessions to either the LDLPFC or RDLPFC, and a sham condition. Decreases for PTSD symptoms were reported for both active conditions, with greater decreases were reported for those in the RDLPFC group. However, in relation to re-experiencing symptoms, equivalent decreases were reported for both active conditions. As such, further investigation of rTMS efficacy, using protocols for MDD, on re-experiencing symptoms and intrusive memories may be of benefit.

Given that meta-analysis has indicated comorbidity rates for MDD and PTSD at 52% [19], an overarching goal of the present study was to capitalise on the reported efficacy of rTMS as a treatment for MDD [14, 15]. This involved assessing established rTMS stimulation parameters for MDD [20] on comorbid traumatic stress symptoms and, more specifically, on the trajectory of intrusive memories given their influence in diagnostic onset and maintenance of PTSD [1, 2]. To the best of our knowledge, there has been no detailed investigation of rTMS influence on intrusive memory frequency or characteristics following trauma exposure.

Literature from the field of memory reconsolidation was also used to inform the investigation of intrusive memory outcomes following rTMS application in the current study. Memory reconsolidation refers to the process whereby a previously consolidated memory is reactivated, thereby rendering it malleable for updating, and then re-stabilised [21–23]. As there is a need to identify interventions that target memory reconsolidation for recovery from PTSD [24], our study drew on memory reconsolidation theories to support extension of current rTMS treatment for MDD, so as to assess its influence on trauma-based memories.

The present study aimed to investigate the role of routine rTMS for MDD, applied in a real-world psychiatric hospital setting, on traumatic stress symptoms overall and, more specifically, on the trajectory of intrusive memory frequency and associated characteristics. In particular, we aimed to investigate the course of change of intrusive memories among people receiving rTMS. No a priori hypotheses were specified in relation to intrusive memory frequency or characteristics, as measured on a daily basis, given the lack of data to date.

Based on previous studies [16–18], we hypothesised that traumatic stress symptoms overall and at the re-experiencing cluster level, as assessed using psychometric tools, would decrease from pre- to post-treatment (Session 1 to Session 20). Decreases for depression were also hypothesised. Due to anticipated decreases in symptoms of traumatic stress and depression, we also hypothesised improvements in symptom severity for the secondary outcome measures of anxiety, stress, sleep, and general mental health.

## Method

### Participants

Participants were 25 adults attending at a private mental health hospital for 20-sessions of rTMS treatment for MDD. Participants were referred for rTMS by their treating psychiatrist. In order to access treatment, and therefore to participate in the study, participants were required to be English speakers over the age of 18. All patients attending for TMS were invited to participate in the research during the study period. Exclusion criteria for participation in this study were consistent with the hospital admission criteria and included nil current problematic substance, psychosis, or active self-harm including suicidal intent. Ethical approval was obtained from the TMS Medical Advisory Committee at the hospital site and the Human Research Ethics Committee at The University of Technology Sydney (ETH19-4019).

### Tasks and Materials

#### **Transcranial Magnetic Stimulation**

Stimulation was applied to the LDLPFC (using the Beam method for F3; [25]) at 10 Hz (4 s trains, 25 s inter-train intervals) using the Magistim D70 AFC coil. The neuronavigation procedure for calibrating the coil location involved identification of the naison and preauricular points for each patient. Resting motor threshold (RMT) was established as the dose at which 50% of the stimuli lead to the finger movement. We noted that less than RMT had significant less finger movement and higher doses lead to greater than 50%, and usually 100% of finger movement. We started at 40%, as this was the average RMT. The depression dose scaling protocol was 1.2 times the RMT.

#### **Interview**

After completing the informed consent process, participants took part in a structured interview that asked about two intrusive memories and their characteristics in a fixed order. The protocol was based on that used by Patel et al. [26] for identification of intrusive memories in patients presenting with major depression. Consistent with memory reconsolidation theories [21–23] and studies investigating traumatic intrusive memories during reconsolidation [27, 28], an essential feature of the current study involved identification of, and elaboration on, two intrusive memories for each participant (memory reactivation) for events that the person’s past that they experienced as distressing. Participants were then asked to provide data on frequency and duration of the memories, and to rate characteristics of the memory (intensity, vividness) and levels of responding (distress, physical sensations, re-experiencing, interference) on a 0–100 scale.

Each memory was given a brief title, which was then used in the individualised survey created for each participant to allow for daily monitoring. The daily questionnaire then served as a reactivation cue for these memories. Participants also completed self-report measures and downloaded the phone app for monitoring of intrusive memories as part of the interview process.

#### **Diary for Intrusive Memory Recording**

Participants downloaded the ExpiWell app [29] on to their Android or IOS phone to track intrusive memory frequency and characteristics daily across Days 0 to 20. Participants monitored intrusive memory frequency over each 24-h period and also reported on associated characteristics using a sliding scale from 0–100. The characteristics monitored included how intense, distressing, and vivid the memories were; along with how much the intrusive memories interfered with the day, sense of being in control when the memories arose, and degree of similarity between emotions and physical sensations experienced when intrusive memories arose and emotions and physical sensations that had occurred at the time of the actual event.

Participants commenced daily monitoring using the phone app on Day 0 using for the duration of the time they were at the hospital receiving rTMS. The app sent an automated reminder each day at 5:00pm to each participant and a follow-up notification was sent if the survey had not been completed within 30-min. Participants ceased daily monitoring of memories once they were discharged from the hospital. The specific questions and corresponding scales are shown in the Supplementary Material.

#### Self-report Measures

##### **Demographics**

Participants completed a questionnaire to obtain age, gender, occupation, first language, medication use, and physical ailment information on Day 0 only.

##### **Brief Trauma Questionnaire (BTQ)**

The BTQ [30] is a 10-item questionnaire that assesses traumatic exposure, consistent with Criterion A of the PTSD diagnostic criteria in the DSM-5 [4].

##### **8-item Posttraumatic Checklist for DSM-5 (PCL8-5) and Re-experiencing Cluster**

The PCL8-5 [31] is an abbreviated version of the 20-item PCL-5 questionnaire. The PCL8-5 is highly correlated with the longer version (*r* = 0.98), has strong internal consistency (Cronbach’s α = 0.93), and is a useful screening tool for PTSD, with scores less than 19 is indicative of diagnostic distress [32]. Good reliability was indicated in the current study, Cronbach’s α = 0.72.

As the PCL8-5 includes only two questions from the re-experiencing cluster of the PCL-5 [33], the additional three items that are included in this cluster were also administered. Strong reliability was indicated for this cluster in the present study, Cronbach’s α = 0.81.

##### **Impact of Events – Revised (IES-R)**

The IES-R [34] 8-item Intrusions subscale was used to assess subjective distress of traumatic events using a 5-point Likert scale (0 = *Not at all* to 4 = *Extremely*) on Days 0,10, and 20 of rTMS. High levels of internal consistency have been reported for this scale, α = 0.86 [35], with similar levels indicated in the current study (Cronbach’s α = 0.88).

##### **Depression, Anxiety, Stress Scale – 21 (DASS-21)**

The 21-item Scale [36] is a self-report measure that uses a 4-point Likert scale (0 = never to 3 = almost always) to assess symptom levels in each domain. Each subscale is comprised of 7-items. High levels of internal consistency have been reported using Cronbach’s α for depression (0.96), anxiety (0.89), and stress (0.93) in large clinical samples, *n* = 678 [37], and non-clinical samples (*n* = 1,794; depression = 0.82, anxiety = 0.90, stress = 0.90) [38]. High levels of reliability (Cronbach’s α) were also reported in the present study for depression (0.88), anxiety (0.84), and stress (0.90).

##### **Montgomery-Asberg Depression Rating Scale (MADRS)**

The MADRS [39] is a 10-item scale used to assess severity of depressive episodes, with higher scores reflecting increased levels of depressive symptoms. Strong internal consistency has been indicated for this scale, Cronbach’s α = 0.84 [40], with good reliability also indicated in the current study (Cronbach’s α = 0.85).

##### **Insomnia Severity Index (ISI)**

The 7-item ISI [41] assesses the patient’s perception of their insomnia over the previous fortnight, with higher scores indicating higher severity of impairment due to insomnia. Strong internal consistency has been demonstrated for the ISI in a primary care setting, Cronbach’s α = 0.84 [42]. Good internal consistency was indicated in the current study (Cronbach’s α = 0.72).

##### **Health of the Nation Outcome Scales (HoNOS)**

The 12-item HoNOS [43] was designed to measure mental health and functioning as reflected by social and behavioural outcomes. Sensitivity to change in inpatient settings has been indicated [44] along with reasonable internal consistency, Cronbach’s α = 0.65 [45]. In the present study, low internal consistency was indicated (Cronbach’s α = 0.42).

##### **Mental Health Questionnaire – 14 (MHQ-14)**

The 14-item MHQ is used to assess the impact of symptoms on general functioning, with higher scores indicating better levels of functioning [46, 47]. Strong internal consistency was indicated in the current study for the total score (Cronbach’s α = 0.84).

### Procedures

After providing informed consent, participants took part in an interview with the researcher and completed routine hospital and study-specific measures. Participants took part in rTMS treatment as usual for 20 sessions and monitored intrusive memories daily over this time. Aside from the demographic and BTQ measures, all other self-report measures were completed on Day 0, at the midpoint of treatment (session 10), post-treatment and at 1-month follow up. Online questionnaires were used to collect data at 1-month follow up, as participants were discharged from the hospital after completing the final rTMS sessions.

### Statistical Analysis

Repeated measures data were analysed using the IBM Statistical Package for Social Sciences (SPSS) version 27. A non-directional alpha level of 0.05 was used for all statistical tests. Repeated measures t-tests were used to determine statistically significant changes between pre and post intervention scores. Consistent with Cohen’s *d* calculations*,* effect sizes were calculated using the sample standard deviation of the mean difference. The reliable change index (RCI) was used to assess change levels beyond the measurement variability range of the measure used and was indicated by an index score of greater than 1.96 of the standard error of the difference [48].

Daily intrusive memory data were analysed and visually represented using SAS (version 9.4, SAS Institute CARY, NC, USA). Frequency of intrusive memories was a zero-inflated count variable with a positively skewed distribution. For this reason, we used generalised linear mixed models for analysis of intrusive memory frequency, with a log link function and negative binomial distribution. General linear mixed models were used for analysis of intrusive memory characteristics. We fitted a) time as a linear term, and b) time included as natural cubic splines to account for non-linearity of change over time, with knots were at the following percentiles: 5, 27.5, 50, 77.5, and 95 [49]. Each participant’s memories were coded as either memory A or memory B, which allowed this variable to be treated as a fixed effect, with individual data at each time point then serving as the repeated measure. The Akaike Information Criterion (AIC; [50]) and Bayesian Information Criterion (BIC, [51]) model fit indices were used for assessment of model fit (between linear and spline terms for time), with smaller values indicating better fit. An exchangeable correlation structure for repeated measurements was used in all analyses with a random intercept for each participant which accounted for individual differences in underlying number of memories and scores for intrusive memories. did not specify a random slope given our small sample size and the importance of a parsimonious model. For the main study outcome (count of intrusive memories) we also estimated a random slopes version of the models with linear and spline terms for time. Model fit was not improved in the random slopes model with linear time (AIC = 3335, BIC = 3342) compared to the random intercept only model (AIC = 3318, BIC = 3327). A random-slopes variant for model with time on a spline basis did not converge despite trying different options for correlation structure and estimation methods.

## Results

### Sample and Demographic Information

Intrusive memory data were analysed for 23 participants (*M*_*age*_ = 48.47 yrs, *SD* = 11.23; females, *n* = 15). Of the 23 participants, 22 reported taking medication for depression specifically and the range of the current episode of depression was reported to be between 7-days through to 55-years.

Comorbid diagnoses were reported for 23 participants, with the most common being anxiety (*n* = 10), PTSD (*n* = 9), and bipolar disorder (*n* = 6). No participants were currently in a manic or hypomanic state at the time of the study. Depressive condition was reported as *chronic* by 14 participants, with the remaining 11 reporting an average of 3.15 years for the current depressive episode.

Each participant endorsed at least one item on the BTQ [26] (*M* = 4.24, *SD* = 2.05) and analysis of the data at an individual level indicated that the trauma memories chosen for monitoring were related to the type of event endorsed on the BTQ.

### Intrusive Memory Outcomes

On average, participants completed 21.83 (*SD* = 6.00) diary entries across the duration of their inpatient admission while receiving TMS treatment. All participant response rates fell within the acceptable range, with no statistical outliers identified. For analysis of intrusive memory characteristics, negative binomial distributions with truncated power basis and a natural cubic spline were used, as this indicated a better fit than linear models. Overall, there was very good evidence in the spline models of an association of time with each characteristic (*p* < 0.05) and AIC and BIC both indicating a better fit with a spline than a linear term (Table 1) for intrusive memory frequency and also the scores of characteristics of the memories.

**Table 1:** Intrusive memory outcomes: Model fit, *p*-values, and change scores Days 1, 7, 20

	AIC	BIC	*p*^*a*^, Day 20	Day 1	Day 7	Day 20	CI 95%, Day 20
Frequency	3345.52	3356.49	<.001	0.77^b^	0.32^b^	0.27^b^	0.18, 0.42^b^
Intensity	4105.43	4116.40	0.004	−3.01	−14.89	−12.97	−21.66, −4.29
Distress	4114.62	4125.59	<.001	−4.02	−17.76	−18.12	−26.89, −9.35
Clear & vivid	4118.64	4129.61	0.004	−3.51	−14.50	−12.96	−21.82, −4.09
Emotion re-experiencing	4167.45	4178.42	<.001	−4.83	−18.61	−17.37	−26.63, −8.11
Physical re-experiencing	4166.00	4176.97	0.020	−2.57	−8.80	−10.93	−20.15, −1.72
Interfered with day	4287.99	4298.96	0.004	−2.55	−11.29	−15.44	−26.04, −4.84
Sense of being in control	4249.19	4260.16	.002	1.96	10.88	16.19	6.05, 26.34

^a^ Comparison to Day 0

^b^ Denotes ratio compared to Day 0

*Note.* For intrusive memory characteristics, score changes compared to Day 0 are indicated at Day 1, Day 7, and Day 20

**Fig. 1:**
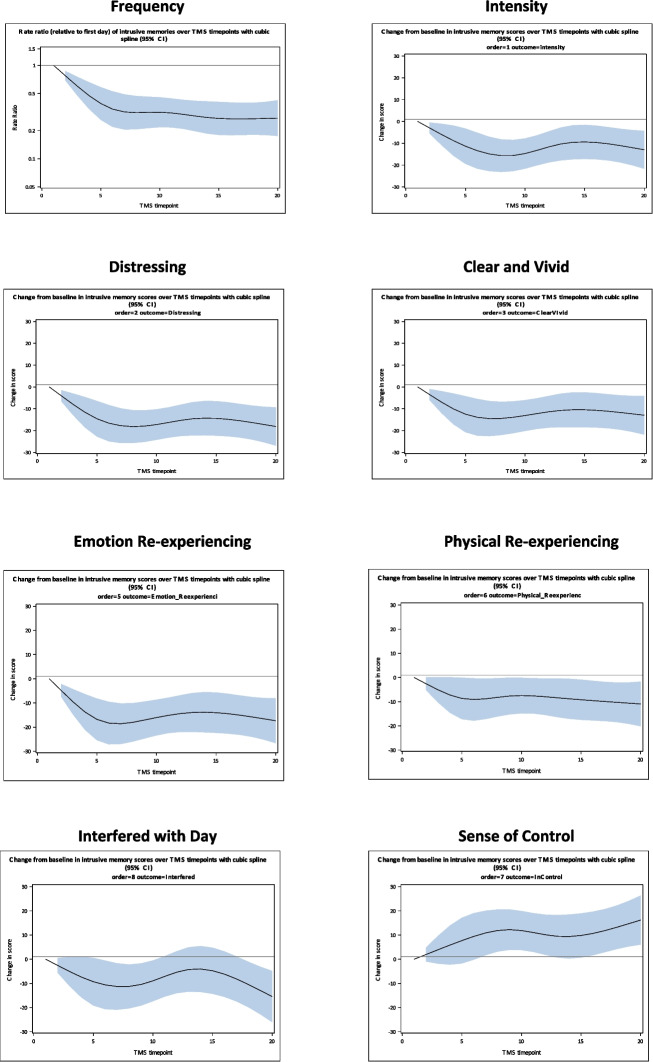
Intrusive memory frequency and characteristic outcomes: Days 1 to 20

For intrusive memory frequency, the pattern was for gradual declines from Days 1 to 7, which were then sustained to Day 20. The first panel in Fig. 1 visually represents the frequency of memories across time as a ratio comparison to Day 0. The shaded area surrounding the trajectory line indicates the 95% confidence interval.

The outcomes for intrusive memory characteristics followed a similar pattern in that decreases reported across Days 1 to 7, were sustained at Day 20, when compared to Day 0. For *sense of being in control,* the pattern is reversed with participants reporting increases across Days 1 to 7, which were sustained through to Day 20. The daily mean score changes are visually represented in Fig. 1, panels 2 to 8, and the statistical outcomes are reported in Table 1.

### Outcomes: Self-report Measures

Decreases were reported for all measures from pre- to post-treatment, except for anxiety. Total MADRS scores decreased from 31.13 (*SD* = 9.00) to 20.00 (*SD* = 12.35; within subjects Cohen’s *d* = 1.06) and total PCL-8 scores (including the additional 3 re-experiencing-related items) decreased from 31.65 (*SD* = 7.15) to 19.32 (*SD* = 12.09), corresponding to a within subjects Cohen’s d value of 1.39. The Pearson’s correlation between change in MADRS and change in PCL-8 (including additional items) from pre to posttreatment was 0.64 (*p* < 0.001). Large effect sizes (> 0.8) were reported for all other measures except insomnia (medium) and anxiety (small). Reliable change was most commonly indicated for traumatic stress symptoms and improvements in general mental health as reflected by the MHQ-14 outcomes. Despite the large effect sizes reported, only half of the sample reported reliable change for the re-experiencing and depression measures. Outcome data for the self-report measures are reported in Table 2.

**Table 2:** Repeated measures outcomes, effect sizes, clinically significant and reliable change for overall sample (*N* = 25)

		Pre-treatment		Mid-treatment		Post-treatment			Significance (*p*)		Effect size Cohen’s *d*		Reliable change (Pre to Post)
Scale	*n*	Mean	*SD*	Mean	*SD*	Mean	*SD*	Pre to Mid	Mid to Post	Pre to Post	Pre to Post	Number	%
PCL8-5	22	24.27	4.61	17.50	7.29	15.05	8.86	** <.001**	.52	** <.001**	1.35	15	68.2
PCL-Re-exp	22	13.14	4.54	10.00	5.10	8.00	5.82	**.002**	.073	** <.001**	1.37	11	50.0
IES-R	22	28.44	7.90	24.62	7.03	20.82	8.63	**.009**	**.017**	** <.001**	1.13	10	45.5
MADRS	24	26.21	7.55	NA	NA	17.79	10.99	NA	NA	** <.001**	0.99	11	45.8
DASS-Depression	25	15.28	4.96	12.48	5.73	10.92	6.26	**.017**	.153	** <.001**	0.90	13	52.0
DASS-Anxiety	25	9.44	5.22	7.96	5.75	7.76	5.13	.064	1.00	.148	0.41	5	20.0
DASS-Stress	25	12.00	5.56	9.76	5.17	8.32	5.46	** <.001**	.256	** <.001**	0.92	9	36.0
ISI	22	17.14	5.29	14.86	7.83	12.77	7.05	.248	.317	**.026**	0.62	5	22.7
HoNOS	20	12.50	4.71	NA	NA	4.70	2.89	NA	NA	** <.001**	1.36	6	30.0
MHQ	22	17.99	11.38	NA	NA	41.92	25.57	NA	NA	** <.001**	−1.12	15	68.2

*Note.* NA = Not administered/applicable; PCL8-5 = Posttraumatic Stress Disorder Checklist for DSM-5; PCL-Re-exp = Re-experiencing cluster, items 1–5 from PCL-5; IES-R = Impact of Events-Re-experiencing subscale; MADRS = Montgomery-Asberg Depression Rating Scale (Items 1–10); DASS = Depression, Anxiety, Stress Scale – 21 items; ISI = Insomnia Severity Index; HoNOS = Health of the Nations Outcome Scale; MHQ = Mental Health Questionnaire – 14 item

### Follow-Up outcomes

For the 1-month follow-up measures, only nine participants completed the online measures. The high rate of attrition did not allow for statistical analyses of the data.

## Discussion

Our study, which investigated the trajectory of trauma-based intrusive memory frequency and associated characteristics in a sample receiving rTMS for MDD, indicated efficacious outcomes of the intervention. Consistent with NICE guidelines [17], participants presenting as inpatients at a psychiatric hospital received 20-sessions of rTMS at 10 Hz applied to the LDLPFC for treatment of MDD. Using principles from memory reconsolidation theories, two target memories were reactivated for each person with participants then monitoring outcomes daily for the duration of treatment [21–23]. Although experience sampling has been used to monitor intrusive memories in a non-clinical sample following the application of rTMS [52], our study is the first to use experience sampling methods to monitor the course of trauma-based intrusive memories within the context of a clinical, inpatient setting for people presenting for rTMS treatment for depression.

Improvements in intrusive memory outcomes were indicated by a pattern in the data that showed consistent decreases for frequency and associated characteristics such as intensity and distress from Days 0 to 7, which were maintained through to Day 20. Participants also reported that the memories became less vivid and that they had less sensory associations (physical and emotional) over time when compared to the initial memories. A similar pattern was seen for sense of control in that there was an increase from Days 0 to Day 7, which was sustained through to Day 20. As agency is associated with positive mental health benefits such as hopefulness and motivation [53], reductions in the symptomatic features of trauma-based intrusive memories may have broader implications for psychological welfare.

Our hypotheses for decreases from pre- to post-treatment for traumatic stress and re-experiencing symptoms were supported, with large effect sizes reported across the course of treatment. These findings are consistent with those from studies that have utilised rTMS protocols akin to those used for MDD to investigate outcomes for PTSD [16–18] and lend further support to this line of investigation. The use of rTMS at 10 Hz is a further strength of the current study given that it had not been previously investigated for influence on traumatic stress symptoms when applied to the LDLPFC.

Interestingly, all participants reported exposure to at least one traumatic event, which was distressing enough to give rise to intrusive memories, with pre-treatment traumatic stress scores being within the diagnostically estimated range for PTSD [32]. The present sample was not selected or screened prior to participation for the presence of trauma-based intrusive memories. Findings for the current study highlight how addressing trauma-focused intrusive memories may have transdiagnostic value and benefit. Targeting traumatic intrusive memories within the purview of routine rTMS treatment for MDD may offer a way to capitalise on its benefits and help to reduce patient and resource burden.

As anticipated, participants reported decreases in depressive symptoms over the course of treatment. The large effect sizes seen are consistent with the extensive literature demonstrating rTMS efficacy for MDD [14, 15]. For the secondary outcomes measured, improvements were demonstrated in relation to stress and general mental health as indicated by large effect sizes, and by a medium effect size for sleep. Our hypothesis in relation to anxiety was not supported with only small effect sizes indicated and decreases from pre- to post-treatment not reaching significance. The lack of significant improvement in anxiety symptoms is consistent with findings from other DLPFC TMS studies. Variability in outcomes may be related to the specific subregion of the DLPFC targeted, as different sites can engage distinct neural circuits involved in anxiety regulation [7].

The high rates of trauma exposure globally [3] coupled with the role of intrusive memories in perpetuating traumatic stress symptoms [1, 2] signal the need for treatments that can target this core feature of PTSD. The emerging body of literature pertaining to rTMS for PTSD indicates benefits, but further studies are needed to establish treatment parameters. Further, the protocols used for PTSD vary from those used for MDD. Our study demonstrates a possible approach is to leverage existing rTMS protocols, as applied for MDD, to reduce intrusive memory and associated pathology following trauma exposure.

### Limitations

Limitations of this study should be considered when interpreting findings and in further investigations. Firstly, our study did not include a control group but instead focused on the course of change for participants by using a within-subjects, daily experience sampling design. As such, further studies involving replication in larger samples with control conditions are needed to ratify findings of the current study. Secondly, despite participants being drawn from a clinical inpatient group, our small sample size limits generalisability of the findings, and outcomes should therefore be interpreted with caution. A further limitation is the absence of sufficient data at follow-up to allow for assessment of gains following discharge. Lastly, although a single researcher who is a registered clinician conducted the interviewer-based assessment of trauma-related intrusive memories, a reliance on self-report measures for other key variables is a limitation. However, the personal nature of intrusive memories necessitated that participants provide information on subjective measures. Future studies may therefore seek to use multi-method assessments such as assessing biological markers or by using observational measures.

## Conclusion

The present study extended on research of rTMS use in traumatic stress and MDD by investigating the trajectory of trauma-based intrusive memories over the course of rTMS application for people attending for treatment of MDD. Strengths of the study included the use of experience sampling methods to eliminate between-subject variance, a careful interviewer-based assessment of the presence and nature of trauma-related memories prior to commencement of rTMS, as well as the application of data analysis approaches which identified non-linear patterns of change in key intrusive memory characteristics. Participants reported significant improvements with large effect sizes over the course of rTMS treatment for intrusive memory frequency and associated characteristics, traumatic stress, and depression. Similar gains were also reported for general psychological welfare. Given that traumatic stress places substantial strain on communities and that mental health systems are already over-burdened [54, 55], it is imperative to find ways to capitalise on existing treatments. Our study demonstrated the clinical utility of attending to traumatic intrusive memories within the context of rTMS for MDD, with outcomes also indicating transdiagnostic gains across several of psychological domains.

## Supplementary Information

Below is the link to the electronic supplementary material.

Supplementary file1 (DOCX 17 KB)

## Data Availability

The data that support the findings of this study are available on request from the corresponding author. The data are not publicly available due to privacy or ethical restrictions.
