# Supplementary material for: Changes in Trauma-based Intrusive Memory Characteristics Associated with Repetitive Transcranial Magnetic Stimulation (rTMS) for Depression: A Daily Diary Study
Source: Psychiatr Q. 2025 May 6;97(1):75–90. doi: 10.1007/s11126-025-10156-4 (PMC13032974; doi:10.1007/s11126-025-10156-4)
Supplement: Supplementary file 1 — Supplementary file1 (DOCX 17 KB) [file 11126_2025_10156_MOESM1_ESM.docx]

Supplementary Material: Diary for Intrusive Memory Recording

Questions:

1. Did you have TMS today?
   - Yes/No
2. How many times did you experience the A intrusive memory in the past 24 hours?
3. How long did the A intrusive memories last each time?
   - 0 to 10 seconds
   - 10 seconds to 1 minute
   - 1 to 10 minutes
   - 10 minutes to 1 hour
   - More than 1 hour
   - Constantly preoccupied
4. How intense were the A intrusive memories?
   - Sliding Scale: 0 = Not at all to 100 = Extremely
5. How distressing were the A intrusive memories?
   - Sliding Scale: 0 = Not at all to 100 = Extremely
6. How clear and vivid were the A intrusive memories?
   - Sliding Scale: 0 = Not at all to 100 = Extremely
7. Did the A intrusive memories seem as though they were happening in the Past, Present/Now, Future?
   - Sliding Scale: Anchored with Past, Present/Now, Future
8. When the A intrusive memories came up, did you re-experience the same/similar emotions to those felt at the time of the actual event?
   - Sliding Scale: 0 = Not at all to 100 = Extremely
9. When the A intrusive memories came up, did you re-experience the same/similar physical sensations to those felt at the time of the actual event?
   - Sliding Scale: 0 = Not at all to 100 = Extremely
10. How in control did you feel when the A intrusive memories came up in the past 24 hours?
    - Sliding Scale: 0 = Not at all to 100 = Extremely
11. How much did the X intrusive memories interfere with your life in the past 24 hours?
    - Sliding Scale: 0 = Not at all to 100 = Extremely

*Notes*

1. *Questions 2-11 repeated for intrusive memory B*
2. *If a participant answer “0” for having experienced the intrusive memory in the past 24-hours, the subsequent questions did not populate for that memory.*
